# Environmental neurodevelopment toxicity from the perspective of Bronfenbrenner’s bioecological model: a case study of toxic metals

**DOI:** 10.1590/0102-311XEN202022

**Published:** 2023-09-18

**Authors:** Homègnon Antonin Ferréol Bah, Nathália Ribeiro dos Santos, Daisy Oliveira Costa, Chrissie Ferreira de Carvalho, Victor Otero Martinez, Erival Amorim Gomes-Júnior, José Antônio

**Affiliations:** 1 Instituto de Saúde Coletiva, Universidade Federal da Bahia, Salvador, Brasil.; 2 Faculdade de Farmácia, Universidade Federal da Bahia, Salvador, Brasil.; 3 Departamento de Psicologia, Universidade Federal de Santa Catarina, Florianópolis, Brasil.

**Keywords:** Maternal and Child Health, Metals, Social Determinants of Health, Child Development, Toxicity, Saúde Materno-Infantil, Metais, Determinantes Sociais da Saúde, Desenvolvimento Infantil, Toxicidade, Salud Materno-Infantil, Metales, Determinantes Sociales de la Salud, Desarrollo Infantil, Toxicidad

## Abstract

A growing body of literature reports the need for an integrated approach to study the effects of the physical environment on the neurodevelopment of children. Assessment of the true neurotoxicity of pollutants cannot be performed separately from the ecological and multidimensional contexts in which they act. In this study, from the perspective of the Bronfenbrenner’s bioecological model, a conceptual model was developed that encompasses the social and biological characteristics of children from the gestational period to childhood, considering exposure to toxic metals. First, we present the toxicity of the main metals and some concept notions that we used in our framework, such as social and structural determinants of health, allostatic load, embodiment, and epigenetic concepts. Then, the main aspects of the Bronfenbrenner’s bioecological model, which allow integration of the gene-social relationship in addition to the physical environment, where these metals act, are explained. Finally, we present and discuss the conceptual framework showing how, in real life, biological and social factors may together influence the neurodevelopment of children. Although this model is based on a group of contaminants, it opens new horizons on how environmental sciences, such as neurotoxicology and environmental epidemiology, can articulate with the theoretical models from human sciences to provide a broader approach to study the effects on human neurodevelopment.

## Abstract (pt)

Uma crescente literatura relata a necessidade de uma abordagem integrada para estudar os efeitos do ambiente físico no neurodesenvolvimento de crianças. A avaliação da verdadeira neurotoxicidade dos poluentes não pode ser realizada separadamente dos contextos ecológicos e multidimensionais em que atuam. Neste estudo, sob a perspectiva do modelo bioecológico de Bronfenbrenner, um modelo conceitual foi desenvolvido para abranger as características sociais e biológicas das crianças desde o período gestacional até a infância, considerando a exposição a metais tóxicos. Primeiro, apresentamos a toxicidade dos principais metais e algumas noções conceituais que utilizamos em nossa abordagem, tais como determinantes sociais e estruturais da saúde, carga alostática, incorporação e conceitos epigenéticos. Em seguida, explicamos os principais aspectos do modelo bioecológico de Bronfenbrenner, que permitem a integração da relação gene-social, além do ambiente físico, onde esses metais atuam. Por fim, apresentamos e discutimos o quadro conceitual mostrando como, na realidade, fatores biológicos e sociais podem, em conjunto, influenciar o neurodesenvolvimento das crianças. Embora esse modelo seja baseado em um grupo de contaminantes, ele abre novos horizontes sobre como as ciências ambientais, como a neurotoxicologia e a epidemiologia ambiental, podem dialogar com os modelos teóricos das ciências humanas para ter uma abordagem mais ampla ao estudar os efeitos no neurodesenvolvimento humano.

## Abstract (es)

Una creciente literatura informa la necesidad de un enfoque integrado para estudiar los efectos del entorno físico en el neurodesarrollo de los niños. La evaluación de la verdadera neurotoxicidad de los contaminantes no puede llevarse a cabo por separado de los contextos ecológicos y multidimensionales en los que actúan. En este estudio, desde la perspectiva del modelo bioecológico de Bronfenbrenner, se desarrolló un modelo conceptual para abarcar las características sociales y biológicas de los niños desde el período gestacional hasta la infancia, considerando la exposición a metales tóxicos. Primero, presentamos la toxicidad de los principales metales y algunas nociones conceptuales que utilizamos en nuestro enfoque, como los determinantes sociales y estructurales de la salud, la carga alostática, la incorporación y los conceptos epigenéticos. A continuación, explicamos los principales aspectos del modelo bioecológico de Bronfenbrenner, que permiten la integración de la relación gen-social, además del entorno físico, donde actúan estos metales. Finalmente, presentamos y discutimos el marco conceptual que muestra cómo, en realidad, los factores biológicos y sociales pueden, en conjunto, influir en el neurodesarrollo de los niños. Aunque este modelo se basa en un grupo de contaminantes, abre nuevos horizontes sobre cómo las ciencias ambientales, como la neurotoxicología y la epidemiología ambiental, pueden dialogar con los modelos teóricos de las ciencias humanas para adoptar un enfoque más amplio al estudiar los efectos sobre el neurodesarrollo humano.

## Introduction

Human development theories help to study multiple perspectives, further the scientific vision about reality, and facilitate communication from the scientific community to decision makers for the application of science to public and social programs. Valuable scientific contributions have been made, helping to shape educational systems and public policies. Some studies focused on specific patterns of neurodevelopment (maturationist theory, Freud’s psychoanalytic theory, and Erik Erikson’s psychosocial development theory), whereas other studies showed a more extended perspective (Piaget’s theory of cognitive development, Vygotsky’s sociocultural perspective, behaviorism, and biopsychosocial theories) ^1^. Bronfenbrenner ^2^ suggested the “ecological model of human development”, a framework with a global approach of social contexts represented as four concentric levels (micro-, meso-, exo-, and macrosystems) in which development occurs. The microsystem is the immediate setting that occurs in everyday life interactions between a growing human being and the components (symbols and people) of that context, such as family and daycare. The interconnection between microsystems is the mesosystem. The exosystem includes settings in which growing human beings are not directly involved (e.g., parents’ occupation) but influence their reality. The macrosystem includes laws, culture, rules, and social norms of a given society. Later, Bronfenbrenner reconceptualized this framework as the “bioecological model of human development”, and included biological and genetic features, showing how the combination of the four settings, by proximal processes, define cognitive, behavioral, learning, and other neurological functions throughout human life ^3^ ^,^ ^4^ ^,^ ^5^. This perspective is “inclusive” and may be applied to other areas involved in human development studies, going this study’s domain.

Recently, scientists have recognized the importance of a holistic approach in environmental sciences. However, they have criticized it for presenting a parallel and separate research with partial perspective from biological and social sciences areas instead of an interdisciplinary perspective. Environmental exposures are socially mediated and do not occur randomly; impoverished communities and non-white populations are frequently the most concerning ^6^ ^,^ ^7^ ^,^ ^8^. According to Senier et al. ^7^ (p. 5), there is a need to “*...follow a path of theoretically-driven science that seeks to link environmental exposures and genetic variants to disease outcomes along precise and multilayered pathways that account not only for biological processes of disease formation, but also consider the social, political, and economic forces that create vulnerabilities to exposure*”.

Some authors have suggested frameworks considering this complexity, such as the exposome concept ^9^ ^,^ ^10^, total environmental ^11^, integrated socio-environmental model of health and well-being ^12^, among others ^8^ ^,^ ^13^ ^,^ ^14^ ^,^ ^15^. Nonetheless, research in this growing and emerging field faces some challenges with the implementation of such a perspective. Senier et al. ^7^ illustrated the fallacy of environmental scientists giving more importance to the biological features even when using the previously mentioned models. They suggested the “socio-exposome” multidimensional framework, which considers the individual, local, and global levels (including sociopolitical conditions) based on the exposome concept ^9^ ^,^ ^10^ and insights from social sciences, public health, and environmental justice activism. Therefore, as an emerging field, there is a need for more commitments to social justice and the use of environmental science expertise in addition to other disciplines to tackle inequalities that maintain the status quo.

To the best of our knowledge, considering the neurodevelopmental toxicity studies in environmental science, very few frameworks ^11^ ^,^ ^13^ encompassing social and biological patterns exist. Ferguson et al. ^16^ reported the situation of limited work on the impact of physical environment on neurodevelopment in the global south and the limits of the methods used in western countries. They suggested the importance of holistic approaches and recommended the use of the Bronfenbrenner bioecological model, extending the ecological concept to the physical environment. For more than a decade, our research group have actively reported on the case of urban and rural communities’ exposure to toxic metals due to industrial and artisanal activities in Bahia, a Northeastern state of Brazil and Latin America ^17^ ^,^ ^18^ ^,^ ^19^ ^,^ ^20^. Based on the literature and our experience as environmental epidemiologists and toxicologists, we suggested a radical shift in the method used to study this topic from the Bronfenbrenner bioecological model perspective due to its capacity to consider the multilevel of human reality, including people’s biology. The colleagues (neuropsychologists and pediatric physical therapists) of our research group provided their expertise along with discussions to establish this framework.

We presented a framework on environmental neurodevelopmental toxicity considering the period from gestation to the second year of life, focusing on early exposure to toxic metals. The first section briefly reviews the metals toxicity and other concepts included in our model. Then, the Bronfenbrenner bioecological model is introduced and these concepts are integrated to show how contaminants and social structure could affect the neurodevelopment of children.

## Important concepts

### Early exposure to toxic metals and child neurodevelopment

Toxic metals are components of diverse chemical forms and their use is the main source of exposure. The threats to human health may be derived from their structures and intrinsic physicochemical, biological, and environmental properties ^21^ ^,^ ^22^. Metals such as lead (Pb), arsenic (As), mercury (Hg), and cadmium (Cd) present no physiological role but are found in humans due to environmental exposure and diet. Women are continuously exposed to these agents ^21^ ^,^ ^22^ ^,^ ^23^ and their biological levels can increase during pregnancy. These contaminants are harmful to the reproductive system and have been associated with several subsequent deleterious effects, including miscarriages and long-term damage to newborns ^23^. Given fetal nutrient demands for growth, many metals may easily cross the placenta and accumulate in fetal tissues, including in the central nervous system (CNS) ^24^.

However, the mechanisms underlying the neurotoxicity of these contaminants remain unclear. Experimental and observational studies have provided valuable information about some plausible pathways ^21^. Pb can interfere with synaptic transmissions and cell adhesion molecules, blocking cell migration during CNS development ^23^. Oxidative stress and cytotoxicity are some of the mechanisms of As neurotoxicity ^25^. The high affinity of Hg for the thiols group may contribute to apoptosis and modulate the cytoskeleton and neuronal receptors ^26^. Exposure to Cd may inhibit the transport of nutrients from mother to fetus, thus causing a drop in anthropometric parameters, explaining neurological abnormalities and developmental delay ^27^. Another toxicity mechanism is the ability of metals to interact with genetic material, leading to dysfunction in the exposed individuals and their descendants ^21^ ^,^ ^24^ ^,^ ^28^. As an example, evidence have shown that As exposure can cause chromosomal aberrations and cellular DNA damage ^25^.

Individual toxic metals have been associated with neurological damage in children after perinatal and childhood exposures. Due to concomitant exposure to multiple pollutants in everyday situations, epidemiological studies have recently focused on the possible interactions between these neurotoxic agents ^29^ ^,^ ^30^. For instance, Pb toxicity was shown to be higher among children with high manganese (Mn) coexposure ^31^ ^,^ ^32^. In addition to the neurotoxic effect of each toxic metal due to prenatal exposure, Freire et al. ^29^ reported a synergistic interactive effect between As and Pb on cognitive performance, whereas the interaction between Mn and Hg was antagonistic.

Furthermore, exposure to metals may be associated with low socioeconomic status or psychosocial stress to disrupt child development ^33^ ^,^ ^34^ ^,^ ^35^ ^,^ ^36^. The possibility of this association makes it necessary to innovate approaches and study their neurotoxicity.

### Social and structural determinants of children’s health and development

The social determinants of health (SDH) are: “*the social, economic, cultural, ethnic or racial, psychological, and behavioral factors that influence the occurrence of health problems and their risk factors in the population*” ^37^ (p. 78).

Despite the SDH being beneficial since its incorporation into practices, according to Crear-Perry et al. ^38^ and Krieger ^39^, to tackle the roots of health inequity, it would be necessary to contextualize them as structural determinants of society that generate social stratification, reflecting the distribution of wealth, power, and privilege. The use of SDH as a simple factor perpetuates the fallacies about health and well-being, particularly at the individual level ^38^.

To illustrate, despite the current social context being favorable for gender equality, in everyday life practice, a child’s well-being and development is mainly associated with women’s health since it is still thought to be a female role ^40^. Therefore, it is crucial to consider the place of women and how they see themselves in society, the oppressions they may suffer according to their skin color, class, other SDHs, and the socio-historical context ^41^. For instance, considering the higher risk of maternal mortality, adverse birth outcomes, and gynecological violence among black women in the United States and Brazil, studies have shown that they simultaneously suffer racism, classism, and gender oppression as the root causes of this situation ^38^ ^,^ ^42^ ^,^ ^43^ ^,^ ^44^. A similar perspective that goes beyond the simple use of SDH as factors is also perceived in the concept of “embodiment” suggested by Krieger ^45^ (p. 225): “*a concept that refers to how we literally incorporate, biologically, the material and social world in which we live, from in utero to death; a corollary is that no aspect of our biology can be understood absent knowledge of history and individual and societal ways of living*”.

These mechanisms involve larger social and historical structures and processes, which create a differentiated distribution of disease processes among groups ^46^. Over time, life circumstances due to social and structural determinants may be embodied by chronic psychosocial stress, causing a wear and tear called “allostatic load”. Biologically, both real threats and the individual’s subjective interpretations of threats can trigger the release of catecholamines, glucocorticoids, and cortisol via the hypothalamic-pituitary-adrenal (HPA) axis ^47^ ^,^ ^48^. These are considered stress hormones that may cross the placenta and impair the fetal CNS development. For example, fetal exposure to maternal cortisol is supposedly regulated by the placenta, which offers partial protection in case of high exposure. Therefore, the allostatic load is thought to be harmful during pregnancy and contributes to the observed disparities in fetal and child health ^14^ ^,^ ^40^ ^,^ ^49^. The quality of mother-infant interactions also has long-term implications for children’s social, emotional, and psychological development ^2^ ^,^ ^40^.

## Bronfenbrenner bioecological model

The Bronfenbrenner bioecological model considers that human development occurs according to one’s immediate setting, biological characteristics, and the structural construct of society. It is a combination of contexts from the distal to the proximal level ^2^ ^,^ ^3^. Here, we will briefly present two properties of our framework ^3^ ^,^ ^4^ ^,^ ^5^.

According to the first property, throughout the life span, human development occurs across processes of reciprocal interactions of growing human being s with people, objects, and symbols in their external environment. This interaction must occur regularly for a sufficient period. Such enduring forms of interaction with the immediate social environment are “proximal processes” that actualize the genetic potential, allowing its expression into phenotypes (e.g., the subject’s psychological and neurological characteristics) ^3^. The model integrates possible non-additive and synergistic effects of the gene-environment relationship; genetic material does not determine neural characteristics but interacts with experiences in the immediate environment. A proximal process is then defined as the transfer of energy between children and the components of their immediate environment. The transfer may occur in one direction or both, separately or simultaneously ^4^. Children’s participation in this process may influence people’s involvement in their growth since, for instance, adults tend to interact more with friendly and smiling children ^50^.

Changes in proximal processes and their quality may contribute to two main types of developmental outcomes: competence and dysfunction. Competence refers to the demonstrated or additional acquisition of skills. For example, children may develop an awareness of their environment and interact with it, control their behavior, and establish and maintain stable relationships. Dysfunction refers to recurrent difficulties in maintaining, controlling, and/or integrating appropriate behaviors and improvements in different developmental domains ^3^ ^,^ ^4^.

**Figure 1:**
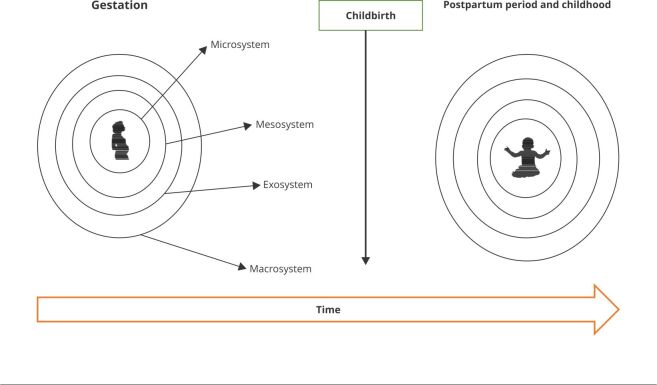
Representation of the four components of the ecological context of child development model considering two periods: during pregnancy and after childbirth.

Source: adapted from Bronfenbrenner ^2^.

According to the second property, the form, power, content, and direction of proximal processes that affect the development vary systematically as a joint function of children’s characteristics and the ecological environment in which these processes occur. It is necessary to consider beyond the proximal processes, the children, the different settings, and the stages they pass over time ^3^ ^,^ ^5^ ^,^ ^51^. The Bronfenbrenner bioecological model conceptualizes the ecological environment as a set of nested systems, ranging from the micro- to macrosystem, changing over time ^2^. The microsystem is composed of a complex set of relationships in the immediate environment of growing human beings (e.g., family and school). The mesosystem represents the relationships between two or more microsystems, in which children actively participate (e.g., the connection between home and school). The exosystem refers to one or more contexts that do not involve growing human beings as active participants but affect their experience in the immediate setting; for example, the parents’ workplace and their friendship networks. The macrosystem refers to the consistencies, in the form and content of lower-order systems that exist or could exist. It describes a system of meanings and customs, including values, attitudes, goals, laws, moral beliefs, and various physical artifacts ^1^ ^,^ ^2^. Figure 1 presents the four components of our model based on the Bronfenbrenner bioecological model.

The chronosystem (not discussed here) includes the socio-historical conditions at the community or society levels. It also includes conditions at the individual level, in which it represents a transition period (e.g., birth of siblings, parents’ divorce). Although not described here, each of these levels carry factors that define the exposure of mothers, fetuses, and children to toxic metals and other contaminants.

## Bioecological model of child neurodevelopmental toxicity due to heavy metals

**Figure 2:**
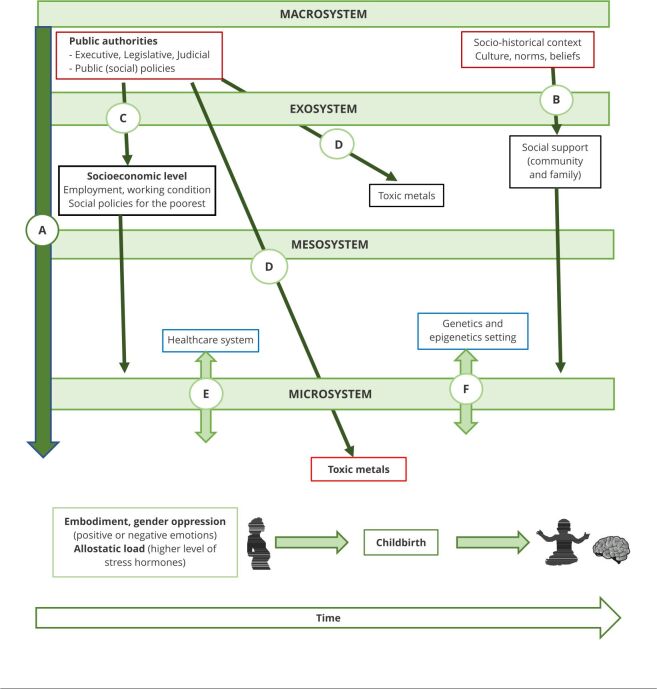
Bioecological framework of early exposure to potentially toxic metals and neurodevelopment.

Source: adapted from Bronfenbrenner ^2^ and Bronfenbrenner & Ceci ^3^. Note: arrow A - shows that the macrosystem defines other systems; arrow B - indicates that the quality of social support mothers’ family receive depends on society perception; arrow C - showing how the macrosystem might define the social context of families, directly and via other systems; arrow D - public authorities can define, regulate, and monitor the exposure to heavy toxic metals of populations; arrow E - shows the relationship between healthcare system and the family setting; arrow F - illustrating the relationship between child immediate setting and gene/epigenetic.

Figure 2 shows some factors that may define exposure to toxic metals and how they could influence the neurodevelopment of children via four components.

### The macrosystem

This level affects people’s lives in other contexts (Figure 2, arrow A); how to educate, feed, and care for a child depends on the culture, geographical situation, among others. It also defines how society perceives pregnancy and the type of support a pregnant woman can receive from her partner, family, and friends (Figure 2, arrow B). Figure 2 (arrow C) represents a set of mechanisms that might (or might not) generate an equitable distribution of resources in the macrosystem according to structural determinant parameters between social classes ^46^. For example, depending on national policies, some countries ensure that the poorest families have access to safe housing, a healthcare system, or a good quality education even if they cannot afford it themselves. However, other countries disregard distributing resources beyond what the individuals can do on their own ^39^.

Moreover, the macrosystem setting includes the possibility of limiting the exposure of populations to potentially toxic metals (Figure 2, arrow D). In this context, legislation-based public institutions may prohibit or monitor the use and management of chemical products and waste ^52^. In Brazil, for instance, the Federal Supreme Court invalidated a labor reform rule that allowed pregnant and breastfeeding women to work in unhealthy activities ^53^. We highlight the role of universities and research institutions that, together with civil society, can pressure public authorities to take adequate measures to protect the population.

### The exosystem

Three parameters may influence a family in the exosystem: employment, social support networks, and the community ^51^.

Work guarantees socioeconomic status, which provides necessary components for children’s development: adequate nutrition, access to healthcare services, and cognitive stimulation. Stress associated with unemployment can impair a couple’s relationship and their physical and mental health, which, in turn, can affect fetuses and children ^51^ ^,^ ^54^. Work is an essential social determinant of mental health ^55^ and can cause unfavorable situations for children development ^51^ ^,^ ^54^. Low-income workers are often exposed to various occupational stressors related to work organizations, such as moral harassments and other forms of violence ^55^. This context might impact the quality of parenting skills at home and, thus, introduce stress into children’s lives ^54^. When considering the physical environment, occupation is the primary exposure factor for toxic metals, exposing pregnant women in unsafe working places. Additionally, secondary exposure may occur when a family member returns home with contaminated clothing or work materials ^23^.

Pregnancy and motherhood are challenging and stressful for women due to changes in their appearance, physiology, emotional well-being, and relationships ^40^ ^,^ ^56^. It usually involves a context of a continuous relationship between two partners, their family, and friends, generating a network of “social support”. Depending on the cultural context, people may express it in several ways (Figure 2, arrow B): physical, emotional, verbal, and financial or assistance to the mother’s self-esteem. This support is important to relieve the physical and emotional stress experienced by mothers. Mothers who had access to social support during pregnancy have reported lower stress, anxiety, and depression, a better marital adjustment, and a more positive attitude towards pregnancy ^40^ ^,^ ^57^. Furthermore, this kind of support favors the adoption of a better lifestyle and facilitates the mother’s return to work ^54^ ^,^ ^56^.

Nevertheless, according to the culture and community level, there are situations in which social support is denied, inadequate, or even harmful, inhibiting children’s growth. For example, one study found that the influence of social support on well-being changed from positive to negative based on three types of factors: low socioeconomic status, occurrence of a tragedy (such as loss of a relative), and doubts about the mother’s ability to take care of herself ^56^. Indeed, pregnant women in adolescent couples depend significantly on the quality of social support (advice and instrumental, informational, and emotional support), which, when inadequate, causes stress that may harm fetuses and/or hinder parents’ relationship with the newborns ^56^ ^,^ ^58^.

### The mesosystem

From pregnancy to childhood, fetuses and children depend on their caregivers. However, the hospital service and the growing human being s’ genetic or epigenetic potential are other settings that affect them ^51^.

Hospital care plays an important role in child development from pregnancy to childhood ^51^ ^,^ ^56^ ^,^ ^58^. Healthcare professionals can detect exposures to toxic metals and support the family by limiting their impact on health (Figure 2, arrow E). They also provide the informational support necessary for mental health and help adopt healthy habits ^58^. The positive impact of hospital care on maternal and child health is greater in an unfavorable social context ^56^ ^,^ ^58^.

Another important aspect is that mesosystem results from the relationship between the genotype and child microsystem (Figure 2, arrow F). The Bronfenbrenner bioecological model hypothesizes that proximal processes are the engine that allows the update and improvement of the genetic potential that each human being is born with ^3^ ^,^ ^51^. Parents transmit genes to their children and provide them with an immediate environment to develop defined characteristics ^50^. However, inadequate proximal processes or exposure to toxic metals may compromise their occurrence by direct effects on DNA or indirect effects via epigenetic processes ^21^ ^,^ ^23^ ^,^ ^24^.

### The family microsystem

**Figure 3:**
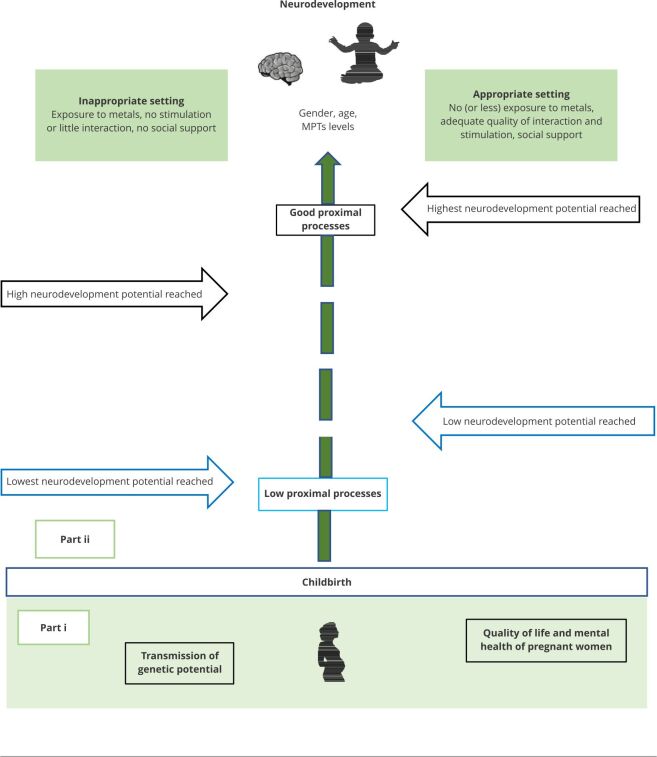
Early exposure to potentially toxic metals and child neurodevelopment in the family microsystem setting.

Inappropriate social processes and exposure to toxic metals can lead to the appearance of vulnerabilities ^35^ ^,^ ^36^ ^,^ ^59^. The changes in the brain induced by stimuli from the microsystem are often referred to as “brain plasticity”. Sensitivity to stimuli, positive or negative, is high during sensitive and rapid growth periods, such as pregnancy and childhood, in which plasticity is greater ^60^. Figure 3 shows the main context of fetuses and children.

MPT: methylparathion. Source: adapted from Bronfenbrenner & Ceci ^3^. Note: Part i - the pregnant woman’s quality of life and (mental) health and her immediate environment allow her to transmit the necessary genetic potential adequately; Part ii - illustrating the influence a child may receive after birth. The central vertical arrow in Figure 3 is dotted to emphasize that the influence of genetics and environment on human development is not separable but is an ever-evolving mixture ^3^; together, they influence the central nervous system by its plasticity ^60^.

#### During pregnancy

During gestation, the microsystem is an intrauterine environment that transmits all the mother’s genetic potential to the fetus. To achieve this correctly, optimum conditions at all ecological levels are required (Figure 3, part i).

However, in real life, a mixture of positive and negative conditions is frequently observed and, depending on the balance between them, a competence or dysfunction might arise ^59^. For example, the mother’s chronic stress due to socioeconomic status may lead to elevated cortisol production, which, after crossing the placenta, may harm the CNS of fetuses, making them more vulnerable since their early stages ^61^ ^,^ ^62^. The release of toxic metals from mothers’ organs put fetuses at risk due to their neurotoxicity. A vicious cycle may be established in which stress and toxic metals impair neuronal processes ^60^ ^,^ ^63^ ^,^ ^64^. Coexposure to psychosocial stress and metals has been associated with a type of toxicity, the mechanism of which might be characterized by interactions. Exposure to stress may decrease the CNS sensitivity threshold to metal toxicity and/or vice versa, affecting neurological functions such as cognition, behavior, and motor function ^35^ ^,^ ^65^ ^,^ ^66^.

#### Postpartum period and childhood

After delivery, children are in direct contact with the physical and social world. However, they still depend on their parents and other adults whose role is to provide the necessary conditions for their growth ^2^ ^,^ ^3^ ^,^ ^4^. Figure 3 (part ii) shows the possible types of the microsystems that children may be exposed to. Although real-life circumstances present many possibilities, for reasons of clarity, we limit ourselves to adequate and inadequate contexts, as suggested by Bronfenbrenner & Ceci ^3^. Children acquire their psychological and cognitive content by a dynamic fusion of the processes of correlations and interactions between the genotype (inherited) and their microsystem ^67^ (represented by the dotted central vertical arrow). The Bronfenbrenner bioecological model considers that proximal processes exert a relatively more powerful effect on development than the environments in which they operate ^3^. Consequently, differences in developmental outcomes between “inadequate versus adequate” environments are consistently smaller than those associated with “low versus high” levels of proximal processes, that is, the quality and strength of interactions between children and their family present a greater impact on neurodevelopment than the type of environment in which they are inserted.

Furthermore, when we extend to the physical environment, several studies showed that children from lower social classes were more sensitive (i.e., loss of IQ points, lower memory capacity) to environmental contaminants (Pb, cigarette smoke, polychlorinated biphenyls, etc.). Simultaneously, those in better conditions were less affected or not affected ^33^ ^,^ ^34^ ^,^ ^36^ ^,^ ^62^. Interpreting this from a bioecological perspective means that the microsystem can provide good conditions and proximal processes that may protect children from the neurotoxic effects of these pollutants. A study ^68^ showed that the social environment (expressed by mothers’ self-esteem) lowered the neurotoxicity of Pb in children. Lucchini et al. ^34^ reported better cognitive performance in children with better social conditions than in others, regardless of exposure to Pb. These results were the same regardless of geographic position ^33^ ^,^ ^34^ ^,^ ^36^. This may confirm the properties of the Bronfenbrenner bioecological model in proximal processes and demonstrate the higher vulnerability of disadvantaged families when exposed to pollutants such as toxic metals.

Activation of the HPA stress-response network has also been suggested as a possible pathway for the neurotoxic effect of Pb ^64^ ^,^ ^67^. For example, Gump et al. ^28^ showed that early exposure to low levels of Pb could alter children’s adrenocortical responses to acute stress, proving the biological plausibility that Pb can also activate the HPA axis. However, we highlight that, among the toxic metals discussed in this study, Pb has been the most investigated regarding its interaction with stress or social context ^62^. Considering other contaminants that have been studied ^35^ ^,^ ^59^ ^,^ ^62^, their coexistence with inappropriate environment causes greater vulnerability in populations.

## Application, strengths, and limitations

Investigating neurodevelopmental toxicity of pollutants involves great responsibility on scientists since their results may influence public policies. Due to the greater vulnerability of growing human being s and developing fetuses, with possible lifetime consequences, it is essential to conduct research adequately. Our framework was based on multiple environmental system perspectives of Bronfenbrenner bioecological model to contribute to current issues discussed in neurotoxicology and environmental sciences ^6^ ^,^ ^7^ ^,^ ^11^ ^,^ ^69^. The radical shift in using social science and humanized approaches makes it unique and different. Considering the need for an environmental science interpreted through the lens of equity, we adapted valuable concepts such as intersectionality (for example, considering the effect of SDH such as gender, race, and social class simultaneously), allostatic stress, and embodiment. Bronfenbrenner bioecological model allows further exploration on pollutants’ impacts on health outcomes and children neurodevelopment, considering individuals, families, and community-level realities over time. The model also offers the opportunity to investigate how determinants at the macrosystem level (like a social policy or legislation) may influence this combined effect (enhancing or mitigating methylparathion - MPT toxicity). Findings obtained with this theoretical model may be more readily accepted and appreciated by the community, civil society, and stakeholders to establish new policies for change.

However, applying this model involves some issues not furthered in this study due to the length restrictions. First, the model includes ecological contexts besides biological (and toxicological) concepts, and it may be expensive to conduct studies that include all aspects of this model. According to Tudge et al. ^70^ ^,^ ^71^, many scholars misuse Bronfenbrenner bioecological model when applying it in their study. Based on analyses of Bronfenbrenner’s scientific production ^2^ ^,^ ^3^ ^,^ ^4^ ^,^ ^5^ ^,^ ^70^ ^,^ ^71^, it appears clear that one can partly use the model. The most important is to clearly define potentially toxic metals (PTMs) exposure, the participants’ characteristics, and the considered and discarded contexts for the investigation. Tudge et al. ^71^ (p. 429) suggested that: “*...appropriate use of Bronfenbrenner’s bioecological theory requires a focus on proximal processes, a means to show that these proximal processes are simultaneously and synergistically influenced both by person characteristics (a minimum of two levels, for example, high and low levels of motivation) and by the context (a minimum of two relevant contexts), and the study should be longitudinal*”.

Therefore, how PTMs exposure, jointly with proximal processes, affects neurodevelopment must be at the center of investigations. For example, a study may consider investigating the effect of exposure to Pb (or MPTs mixtures) on children’s cognition at two moments (after six months and two years), by comparing girls vs. boys (or low birth weight vs. normal birth weight) in two socioeconomic contexts; by the proximal processes being assessed by mother-child interaction quality using the *Home Observation for Measurement of the Environment* (HOME) questionnaire; or using other tools that evaluate the degree of interaction and stimulation from family members.

A second issue to consider is the assessment of MPTs (or pollutants) exposure and other biological parameters (genetic, immunologic, microbiome), which may be considered in the interplay between MPTs neurotoxicity and ecological aspects. For example, questionnaires, spatial distribution, biomarkers (blood, nails, saliva, hair), environmental sample (water, dust, air, soil), sensors, and “omics” technologies are some tools that may be helpful. However, as alerted by Siroux et al. ^15^, a caution should be considered to avoid misclassification.

A third important issue is analyzing and interpreting large-scale and diverse data. Indeed, applying this multilevel framework involves statistical challenges. Separate regression analyses, multilevel modeling, nonparametric smoothing methods (such as locally weighted polynomial regression), and structural equation modeling are examples of statistical methods applied in literature ^8^ ^,^ ^69^ ^,^ ^71^ to estimate the joint effect of exposures and or ecological variables on human health. The choice of the adapted statistical tools should be well explained by the investigator to contribute critically and help to improve this theoretical model.

## Conclusion

Biological sciences, such as toxicology, have allowed important advances in society, helping to understand and improve life. Although scientists practice with the intent of neutrality, in recent decades, the importance of including “what is experienced and resentful” by participants in research has been demonstrated to better approach reality. Concepts such as the “embodiment” of the social context, the allostatic load, and SDHs express this more accurately. Regarding human development, the Bronfenbrenner bioecological model has integrated several areas. Our theoretical model has attempted to assess the impact of contaminants, such as toxic metals on the neurodevelopment of children, suggesting explorations of new paths. Despite the presence of an extended period between the research results and the concrete political decisions for social justice, there is an urgent need for more humanized research that puts vulnerable populations at the heart of the scientific investigation.
